# Effect of different freezing rates during cryopreservation of rat mesenchymal stem cells using combinations of hydroxyethyl starch and dimethylsulfoxide

**DOI:** 10.1186/1472-6750-12-49

**Published:** 2012-08-13

**Authors:** Yahaira Naaldijk, Marek Staude, Viktoriya Fedorova, Alexandra Stolzing

**Affiliations:** 1 Fraunhofer Institute for Cell Therapy and Immunology, Perlickstrasse 1, Leipzig, 04103, Germany

**Keywords:** Mesenchymal stem cells, Cryopreservation, Controlled rate freezing, Hydroxyethyl starch

## Abstract

**Background:**

Mesenchymal stem cells (MSCs) are increasingly used as therapeutic agents as well as research tools in regenerative medicine. Development of technologies which allow storing and banking of MSC with minimal loss of cell viability, differentiation capacity, and function is required for clinical and research applications. Cryopreservation is the most effective way to preserve cells long term, but it involves potentially cytotoxic compounds and processing steps. Here, we investigate the effect of decreasing dimethyl sulfoxide (DMSO) concentrations in cryosolution by substituting with hydroxyethyl starch (HES) of different molecular weights using different freezing rates. Post-thaw viability, phenotype and osteogenic differentiation capacity of MSCs were analysed.

**Results:**

The study confirms that, for rat MSC, cryopreservation effects need to be assessed some time after, rather than immediately after thawing. MSCs cryopreserved with HES maintain their characteristic cell surface marker expression as well as the osteogenic, adipogenic and chondrogenic differentiation potential. HES alone does not provide sufficient cryoprotection for rat MSCs, but provides good cryoprotection in combination with DMSO, permitting the DMSO content to be reduced to 5%. There are indications that such a combination would seem useful not just for the clinical disadvantages of DMSO but also based on a tendency for reduced osteogenic differentiation capacity of rat MSC cryopreserved with high DMSO concentration. HES molecular weight appears to play only a minor role in its capacity to act as a cryopreservation solution for MSC. The use of a ‘straight freeze’ protocol is no less effective in maintaining post-thaw viability of MSC compared to controlled rate freezing methods.

**Conclusion:**

A 5% DMSO / 5% HES solution cryopreservation solution using a ‘straight freeze’ approach can be recommended for rat MSC.

## Background

Mesenchymal stem cells (MSC) provide a useful tool for regenerative medicine due to their differentiation capacity, immunosuppressive properties, secretome profile and migratory ability [1,2].

MSC represent a valuable source for research and clinical applications due to their ability to produce a range of different cell types including osteoblasts, adipocytes, chondrocytes and myoblasts [3-6]. Effective cryopreservation MSCs offers an opportunity to advance the potential use and implementation of these cells into clinical applications.

Cryopreservation itself can affect differentiation capacity of stem cells [7,8]. The loss of a variety of pluripotency markers has been associated with cryopreservation [9,10] but the precise reasons for these changes remain to be explored.

Many studies on the cryopreservation of MSCs were carried out using slow-rate cooling methods [11,12] which is often considered a superior method of preservation [13,14]. However, limited evidence exists whether the freezing rate in fact affects stem cell growth and differentiation potential. Both ‘slow’ [15-19] and ‘fast’ [20,21] freezing protocols have reported ‘success’ as far as maintaining similar phenotypes, cell surface markers and growth rates in comparison with unfrozen MSC.

Several groups have investigated MSC (from different sources) cryopreservation using 10% DMSO and slow freezing protocols. In these studies, the cryopreserved MSC maintained similar phenotypes, cell surface markers and growth rates in comparison with fresh cells [19,22,23]. In addition fast freezing protocols (vitrification) have been investigated with MSC, showing normal proliferation, phenotype and differentiation [20,21].

To facilitate freezing, a cryoprotectant is usually added. An ideal cryoprotection solution should be nontoxic for cells and patients, nonantigenic, chemically inert, provide high survival rate after thawing and allow transplantation without washing. The most commonly used cryoprotector, DMSO, shows cytotoxicity [7,8]. Clinically, DMSO can cause leukoencephalopathy [24], epileptic seizures [25] or elevated lactate dehydrogenase levels [26] after transplantation of DMSO-preserved human bone marrow cells. DMSO is thought to interact with the metabolism and membrane of cells, resulting in cell damage [27]. Nonetheless, DMSO is widely seen as indispensible at least as a component of a cryoprotectants solution.

Additions to DMSO include methycellulose [28], PVP [29], trehalose [30] or others and these components are not investigated here.

Another substitution compound for DMSO in cryoprotection is Hydroxylethyl Starch (HES) which is used in the clinical setting as a plasma volume expander [31-35]. A number of different cell types have been cryopreserved using HES [36-38] with red blood cells being routinely cryopreserved in cryoprotective solutions containing HES [33,39,40]. Bone marrow cells from human and other animal species have also been cryopreserved in HES-containing solutions [41-43].

Physical and chemical properties such as solubility, molecular stability as well as rate of hydrolysis and metabolism depend on molecular weight (MW) and degree of substitution of HES molecules. HESs with lower MW have higher solubility and slower breakdown rates [41,43]. A variety of different HESs with different MW are currently clinically approved and commercially available.

In this study we attempt an initial comparison of two independent factors: freezing rates and cryopreservation solutions. We analysed their effect on viability, growth characteristics and differentiation potential of rat MSCs after cryopreservation.

## Methods

### Isolation of mesenchymal stem cells

The rat (Sprague Dawley, 2–3 month old, male) was killed by controlled inhalation of CO_2_. The hind legs were removed, the soft tissue removed and the separated bones (tibia, femur) were stored in PBS. The bones were centrifuged at 1000 rpm for 5 min and bone marrow was resuspended in 1 ml DMEM.

### Cell culture

Cells obtained from each animal were distributed into two T75 flasks and incubated at 37°C. The first medium change was done after 5 days and afterwards every 2 to 3 days. For the experiments we used MSCs from passage 1 to passage 3.

Rat MSCs were cultured in 1x Dulbecco’s modified Eagle’s medium (DMEM, 1 g/L D-Glucose, Invitrogen) containing 10% fetal bovine serum (FBS, Hyclone) and 1% penicillin/streptomycin (pen/strep, Invitrogen).

### Cryopreservation

MSCs were frozen when they reached 80% confluency. Prior to freezing, cell number was determined by trypan blue staining using a Neubauer hemocytometer. 10^5^ cells were added to each cryogenic vial (2 ml Nalgene). MSC were centrifuged for 5 min (1000 rpm at room temperature) to pellet the cells, media was removed and CPA was slowly (10 sec) added with a pipette and the cells were carefully re-suspended. CPA were containing 500 μl of prepared cryoprotectant consisting of hydroxyethyl starches of different mean molecular weights (MW = 109, 209, 309, 409, 509, 609 kDa - Serumwerk, Bernburg) and/or DMSO (Sigma-Aldrich)). Cryogenic vials were kept on ice until samples were frozen applying the different freezing protocols (Table 1) using a rate controlled freezing system (Thermo Scientific) where indicated Model 7452 Series). Protocols used on this study were modified or newly designed based on published protocols [35,44-46]. The chamber of the freezing system was pre-cooled to 4°C before each experiment. The samples were stored at −134°C in the vapor phase segment of a liquid nitrogen tank for at least 24 h. Samples were thawed at 37°C in a water bath and 10^4^ cells were seeded per well in triplicates into pre-warmed culture medium. Fresh unfrozen MSC were seeded as a control.

**Table 1:** Freezing protocols used

**Protocol**	**Description**	**Duration [min]**	**References**
1	0,3°C/min to −100°C	347	
	Store in vapour phase at −134°C		[47]*
2	1°C/min to −80°C	84	[46-50]
	Store in vapour phase at −134°C		
3	1°C/min to - 30°C	44	
	5°C/min to −80°C		[44,51]*
	xStore in vapour phase at −134°C		
4	1°C/min to −20°C	34	
	5°C/min to −40°C		
	10°C/min to −80°C		Designed
	20°C/min to −100°C		
	Store in vapour phase at −134°C		
5	1°C/min to −6°C	26	
	25°C/min to −50°C		Designed
	10°C/min to −90°C		
	Store in vapour phase at −134°C		
6	Directly into the vapour phase	0	
7	99°C/min to −100°C	2	
	Store in vapour phase at −134°C		[35,52]*

* Modified.

### MTT assay

The MTT assay was performed 3 days after thawing. Each well was filled with 500 μl of media containing of MTT-reagent, consisting of 5 mg/ml MTT (Carl Roth) in PBS. After incubation for 4 h at 37°C, medium was removed and 500 μl stop-solution (10% SDS (Merck) and 50% dimethylformamide, (VWR International)) was added. The cells were incubated overnight at 37°C and absorbance was measured using a microplate reader (TECAN) at 550 nm and 630 nm as reference wavelength.

### Osteogenic differentiation

The day after thawing medium was changed to osteoinductive medium (low Glucose DMEM; 10% FBS; 1% pen/strep; 10 nm dexamethasone, (Sigma-Aldrich); 50 μg/ml ascorbic acid 2-phosphate, (Sigma-Aldrich)). Differentiation media was changed every 2 days for a period of 14 days. For qualitative analysis of osteogenic differentiation, cells were fixed in 70% ethanol for 15 min and washed once with ddH2O.

After washing, cells were stained with ALP buffer pH 8.5 (0.2 M Tris, 1 mg/ml fast red, Sigma and 50 μg/ml naphtol phosphate AS-BI, Sigma) for 1 hr.

### Adipogenic differentiation

Adipogenic medium (10% FBS; 1% pen/strep, 10% insulin-transferrin-selenium supplement, (Sigma-Aldrich) 10^-8^ M dexamethasone (Sigma-Aldrich); 0.5 mM isobutylmethylxanthine, Sigma-Aldrich; 100 μM indomethacin, Sigma-Aldrich) was added the day after thawing. The media was changed every 2 days. After 14 days cell phenotype was analyzed by Oil Red O (Sigma-Aldrich) staining.

### Chondrogenic differentiation

Chondrogenic media (10% FBS; 1% pen/strep 1% insulin-transferrin-selenium supplement (Sigma-Aldrich), 10-7 M dexamethasone (Sigma-Aldrich), 150 μM ascorbic-2-phosphate (Sigma-Aldrich), 20 μM linoic acid (Sigma-Aldrich) and 0.1 ng/ml TGF-β (Oncogenic Sciences) was added after thawing. After 2 weeks, cells were stained with Alcian Blue (Sigma-Aldrich).

#### Quantitative alkaline phosphatase (ALP) assay

After 14 days of differentiation, the 24 well-plates were washed and fixed with ice-cold 70% ethanol for 20 min. Ethanol was removed, the plates washed and incubated with 1 ml of p-nitrophenylphosphate (1 mg/ml, Calbiochem) in TRIS (pH 8.0). Cells were incubated for 1 h at RT and absorbance measured at 405 nm using a microplate reader (TECAN). Subsequently, the 24 well-plates were rinsed with ddH_2_O and washed with 10 mm borate buffer (pH 8.5, Sigma-Aldrich). 500 μl methylene blue (1 mg/ml, Sigma-Aldrich) in 10 mm borate buffer was added to each well. After 30 min incubation at RT the plates were washed with 10 mm borate buffer and 500 μl 1% hydrochloric acid (VWR International) was used for dye elution. Plates were incubated for 30 min at RT and absorbance was measured at 650 nm using a microplate reader (TECAN).

#### Phenotyping of mesenchymal stem cells

Cells were incubated with CD90 (1:50, Abcam), CD45 (1:100, AbD Serotec), CD11b (1:100, Abcam), and CD44 (1:50, Millipore) for 1 h at 4°C, washed and incubated with Cy2 (1:750, Jackson ImmunoResearch) for 45 min at 4°C. Cells were washed again and analyzed using the Cytomics FC500 flow cytometer and CXP Analysis 2.1 software (Beckman Coulter).

#### Cell morphology

Morphology of the cells was analyzed 3 days after thawing by light microscopy. Pictures were taken at 10x magnification.

#### Statistics

All experiments were repeated at least three times. Statistical analysis was performed using ANOVA followed by Turkey test, with p < 0.05 considered an indicator of robustness (although not of absolute statistical significance as the number of experiments was too low).

## Results

Rat MSCs were cryopreserved in vials using seven different freezing protocols (Table 1). Different concentrations of DMSO and HES alone or in mixture were tested.

### Verification of consistent nucleation

Some studies (in the 80's) [53,54] have stressed the importance of capturing a consistent ice nucleation point. While in our experiments (in keeping with many machine-based freezing practices in tissue engineering) nucleation was not initiated ‘manually’, we can show that nucleation occurs consistently as indicated by a characteristic [55] ‘heat release’ spike (Additional file 1 Figure S4).

### Post-thaw phenotyping

**Figure 1:**
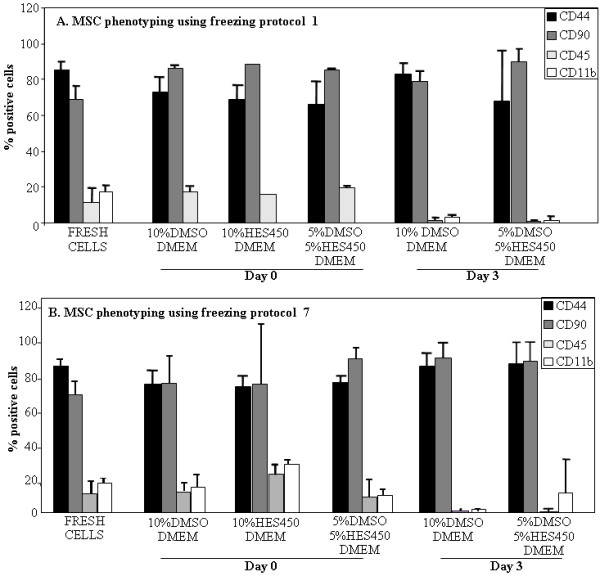
**MSC phenotyping.** Level of CD-expression in MSCs cryopreserved in different solutions directly after thawing (day 0) and after 3 days with protocol 1 (**A**) and protocol 7 (**B**).

CD90, CD44, CD45 and CD11b expression were measured in samples cryopreserved using protocol 1 and 7, in 90% DMEM and three different cryosolutions: 10% DMSO, 10% HES 450 and 5% DMSO/5% HES 450. CDs were measured directly after thawing and 3 days later and compared to non-cryopreserved cells (Figure 1). As expected, both MSCs after cryopreservation and non-cryopreserved MSCs have low expression of hematopoietic stem cell markers CD45 and CD11b and high expression of mesenchymal-associated marker CD90 and CD44 [21,56,57]. No differences in CD expression were observed between day 0 and 3 in both protocols. Usage of HES 450 and DMEM results in low cell number after 3 days therefore no CD phenotyping was performed.

### Post-thaw viability

**Figure 2:**
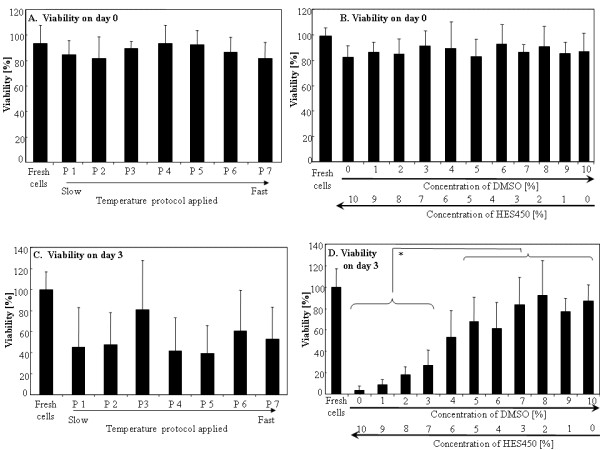
**The effect of cryopreservation on MSC viability immediately after thawing and after 3 days at various HES/DMSO combinations and freezing rates.** Viability of MSCs after cryopreservation with either different freezing rates (**A**, **C**) or different concentrations of DMSO and HES 450 (**B**,**D**) measured directly after thawing (**A**, **B**) and after 3 days by MTT (**C**, **D**).

**Figure 3:**
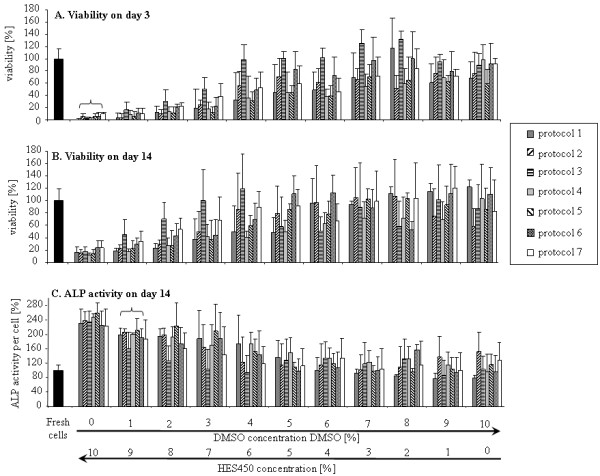
**Cryopreservation of MSCs using HES/DMSO combinations.** Viability of MSCs after cryopreservation with different concentrations of DMSO and HES 450, measured by 3 days after thawing in normal media (**A**) and after 14 days of osteogenic differentiation (**B**). Alkaline phosphatase activity of cryopreserved MSCs after osteogenic differentiation for 14 days (**C**).

Directly after thawing, we recorded approximately 85% cell viability with no observed difference between the different protocols and cryoprotectant-solutions (Figure 2A-B). However, viability of cells directly after thawing cannot represent reliable criteria for estimation of cryopreservation efficacy. After cultivation for 3 days, a considerable decrease in viability for some solutions was observed (Figure 2C-D). This post-thaw decrease in cell viability is known to be related to apoptotic and necrotic processes which occur within first 24 hours and are not evident immediately after thawing [16,58,59]. On day 3 after thawing, DMSO concentrations under 4% are associated with reduced MSC viability (Figure 2D). The solution of 8% DMSO / 2% HES 450 shows on average of all protocols the highest viability compared to all solutions with 5% and less DMSO. Reliance on HES alone (10% HES 450) is associated with low cell viability. MSC viability was maintained at 14 days (Figure 3B) showing that proliferation activity is not affected by prolonged culture (Additional file 2 Figure S2).

**Figure 4:**
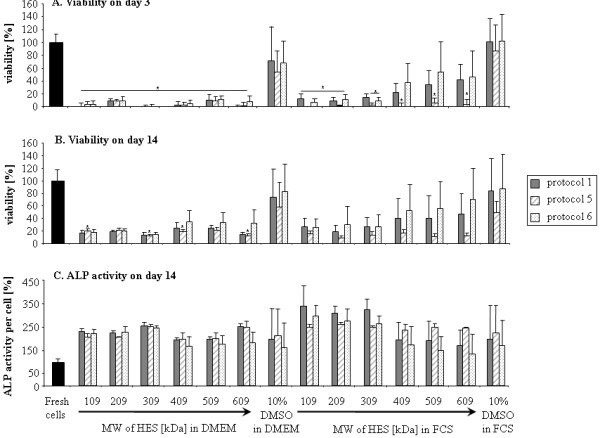
**Effect of HES with different molecular weight distributions on MSCs cryopreservation.** Viability of MSCs after cryopreservation with 10% HES of different molecular weight distributions (109–609) in combination with DMEM or FCS, measured 3 days after thawing (**A**) and by methylene blue staining after osteogenic differentiation for 14 days (**B**). Alkaline phosphatase activity of cryopreserved MSCs after osteogenic differentiation for 14 days (**C**).

HES with the same molar substitution (0.7) but different molecular weight distributions were investigated in order to determine the relation between size of HES and its cryoprotective capacity. We used the cooling protocols 1, 5, and 6 (Table 1) which were chosen as the protocols with the most different parameters. No difference in cell viability at day 0 was observed between the solutions and protocols (data not shown). HES solutions in DMEM and FCS showed less cell viability at day 3 post-thawed compared to DMSO controls (Figure 4A).

As a trend, higher HES molecular weight seems to sustain cell viability (with notable exception for protocol 5) but only in FCS not in DMEM (Figure 4A and B).

### Post-thaw differentiation capacity

Thawed MSC retain their capacity to differentiate toward osteoblasts, adipocytes and chondrocytes. Qualitative assessment of the osteogenic, adipogenic and chondrogenic differentiation of MSC shows no difference in effect between the different cryosolutions (Additional file 3 Figure S1), but we only pursued osteogenic capacity in greater detail.

ALP activity is lower at ‘high’ (>5%) levels of DMSO compared to solutions with a higher HES 450 content (Figure 3, C and Additional file 4 Figure S3).

No differences could be observed between HES of different molecular weights and there was no effect attributable to concurrent use of either serum or DMEM (Figure 4c).

### Post-thaw phenotyping

CD90, CD44, CD45 and CD11b expression were measured in samples cryopreserved using protocol 3 and 6, in three different cryosolutions: 10% DMSO + medium, 10% HES 450 + medium and 5% DMSO/5% HES 450 + medium. CDs were measured directly after thawing and 3 days later and compared to non-cryopreserved MSC (Figure 1).

As expected, both MSCs after cryopreservation and non-cryopreserved MSCs have low expression of hematopoietic stem cell markers CD11b and CD45 and high expression of mesenchymal-associated marker CD90 and CD44 [56,57]. No differences in CD expression were observed between day 0 and 3 in both protocols. Cryopreservation did lead to a further reduction of hematopoetic makers CD11b and CD45. Usage of HES 450 and DMEM results in such low cell number after 3 days that no CD phenotyping was possible.

## Discussion

It is not uncommon to analyze cell viability directly after thawing [47,48,60-62], however our results clearly show that this is an inaccurate measure of cryopreservation effects in rat MSC, since the different effects of various cryosolutions tested were only observable 3 days after thawing and beyond. Cryopreservation associated cell death is known to occur from 6 h after thawing and beyond [63-66]. We investigated different cooling protocols and found a constant cooling rate of 1°C per min until −30°C followed by 5°C per min until −80°C and protocols with a fast cooling rate (protocols 1 & 6) are equally suitable considering cell viability and recovery in the cryopreservation of rat MSCs. However, the choice of protocol seems to have a rather marginal effect on post-thaw viability, with little difference to a ‘straight freeze’ approach (protocols 6) that saves considerable time and effort. Survival rates were in the range of previous reports and MSC phenotypes were not affected by cryopreservation. It could be argued that protocols 1–3 are essential ‘the same’ with regards to the pre-cooling rate of hypothetical importance, i.e., the one that governs the biophysical response of the cells being cooled. Indeed, we observe no difference between these protocols. However, since the different protocols are in practical use we have maintained their differentiated profile for reference.

### DMSO reduction using alternative cryoprotectants

As discussed above and previously [43], the use of DMSO has several disadvantages. However, based on these results in rat MSC, a total substitution of DMSO with HES is not advisable if cell viability is a key indicator. Upon reducing DMSO concentration below 4% an observed decrease in cell viability can be measured, probably because of ice crystal growth in the relatively large cell body of the MSC and reduced osmotic exchange of water against DMSO during the relatively short incubation time of the cells in DMSO.

However, we could observe a slight reduction of osteogenic capacity related to higher DMSO concentrations. It is known that oxidative stress induces differentiation [67], which also occurs during freezing [68]. A badly designed freezing protocol might induce differentiation impacting on the quality of the stem cells.

If these or other drawbacks of DMSO are a factor, our results show that a partial replacement of DMSO with HES is certainly possible. Studies using a 6% HES + 5% DMSO solution usually show superior cryopreservation in comparison to 10% DMSO [43]. Regarding the ‘optimum’ between MSC survival and osteogenic differentiation one can conclude that while lowest DMSO concentration are slightly better for differentiation for practical tissue engineering purposes a DMSO concentration of 5% or slightly higher should be preferable.

#### Molecular weight of HES

In previous cryopreservation studies different MW of HES ranging from 150 to 450 kDa were used but not compared, making it difficult to know if there are differences between the variable HES solutions [33,69]

For the first time we compared the effects of HES ranging from 109 to 609 kDa with a similar hydroxyl substitution rate on viability and osteogenic differentiation. The cryopreservation with HES of different molecular weights had no effect on survival and differentiation of MSCs.

## Conclusion

1. The study confirms that, for rat MSC, cryopreservation effects need to be assessed some time after, rather than immediately after thawing.

2. MSCs cryopreserved with HES maintain their characteristic cell surface marker expression as well as the osteogenic, adipogenic and chondrogenic differentiation potential.

3. There are no major changes in the expression of surface proteins identifying MSC, proliferation capacity and osteogenic differentiation in MSC frozen with 5% DMSO/ 5% HES.

4. HES alone does not provide sufficient cryoprotection for rat MSCs, but provides good cryoprotection in combination with DMSO, permitting the DMSO content to be reduced to 5%. There are indications that such a combination would seem useful not just for the clinical disadvantages of DMSO but also based on a tendency for reduced osteogenic differentiation capacity in rat MSC cryopreserved with high DMSO concentration.

5. HES molecular weight appears to play only a minor role in its capacity to act as a cryopreservation solution for MSC.

6. The use of a ‘straight freeze’ protocol is no less effective in maintaining post-thaw viability of MSC compared to controlled rate freezing methods.

As a simplified summary, a 5% DMSO / 5% HES solution cryopreservation solution using a ‘straight freeze’ approach can be recommended as ‘optimal’ for ‘normal’ rat MSC cryopreservation.

## Competing interests

This work was supported in part by Serumwerke Bernburg a manufacturer of HES.

## Authors’ contributions

MS carried out the bulk of the experiments, VF contributed to some phenotyping experiments and sections of the manuscript. YN conducted the phenotyping and chondrocyte experiments, consolidated data and provided the first draft of the manuscript. AS planned the experiments, secured the funding and revised the manuscript. All authors read and approved the final manuscript.

## Supplementary Material

Additional file 1 **Figure S4.** Freezing curves of the machine cryopreservation protocols. The curves for the machine based freezing rates were recorded by the machine and summarized here for the protocols 1–6. The curves show just small variations during the heat release phase between 0 and −10°C. Click here for file

Additional file 2 **Figure S2.** Cellular Morphology of cryopreserved rat MSC. Morphology of cryopreserved rat MSC after 14 days in culture. Click here for file

Additional file 3 **Figure S1.** Differentiation capacity of rat MSCs after cryopreservation. Qualitative ALP-staining of MSCs after 14 days osteogenic differentiation. Magnification 20X (**A**). Oil red O staining of MSCs after 14 days in adipogenic differentiation medium. Magnification 40X (**B**). Chondrogenic staining of differentiated MSCs cells after 14 days. Magnification 20X (**C**). Click here for file

Additional file 4 **Figure S3.** Morphology of cryopreserved rat MSC during osteogenesis. Cellular morphology of cryopreserved rat MSC after 14 days in osteogenic differentiation medium. Click here for file
