# Supplementary figures and images for: Effect of different freezing rates during cryopreservation of rat mesenchymal stem cells using combinations of hydroxyethyl starch and dimethylsulfoxide
Source: BMC Biotechnol. 2012 Aug 13;12:49. doi: 10.1186/1472-6750-12-49 (PMC3465236; doi:10.1186/1472-6750-12-49)

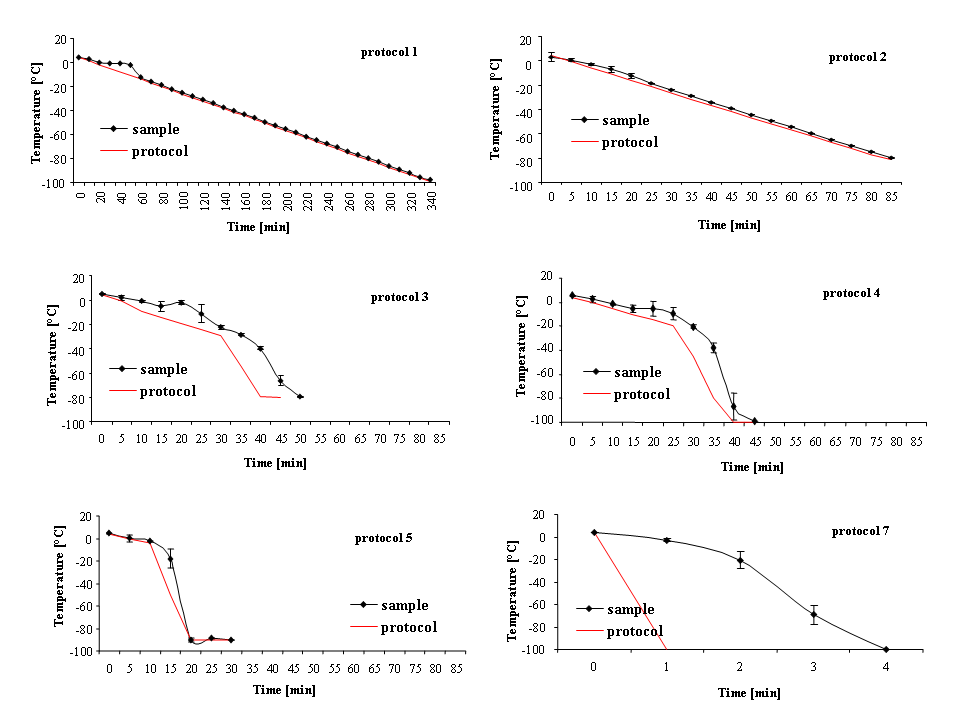

Supplement: Additional file 1 — Figure S4. Freezing curves of the machine cryopreservation protocols. The curves for the machine based freezing rates were recorded by the machine and summarized here for the protocols 1–6. The curves show just small variations during the heat release phase between 0 and −10°C. [file 1472-6750-12-49-S1.tiff]

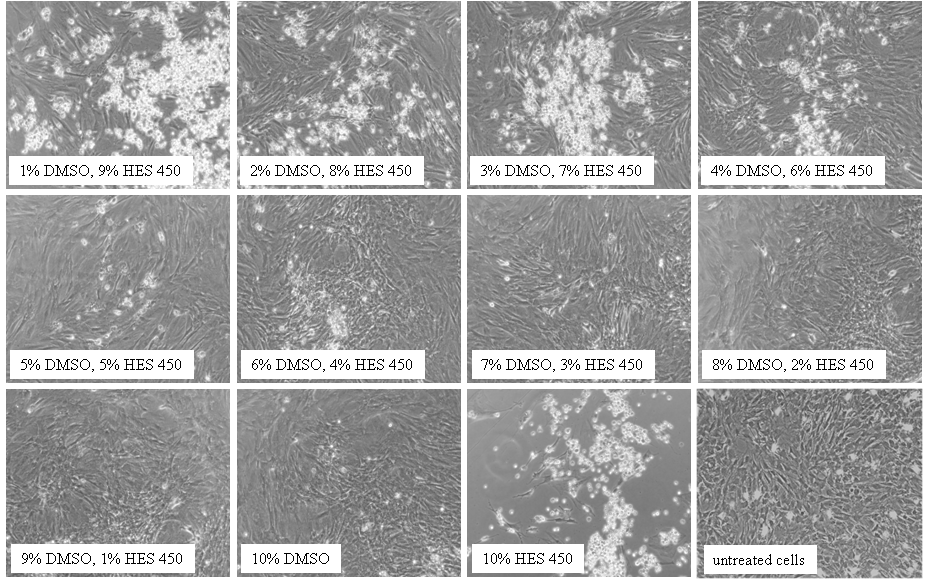

Supplement: Additional file 2 — Figure S2. Cellular Morphology of cryopreserved rat MSC. Morphology of cryopreserved rat MSC after 14 days in culture. [file 1472-6750-12-49-S2.tiff]

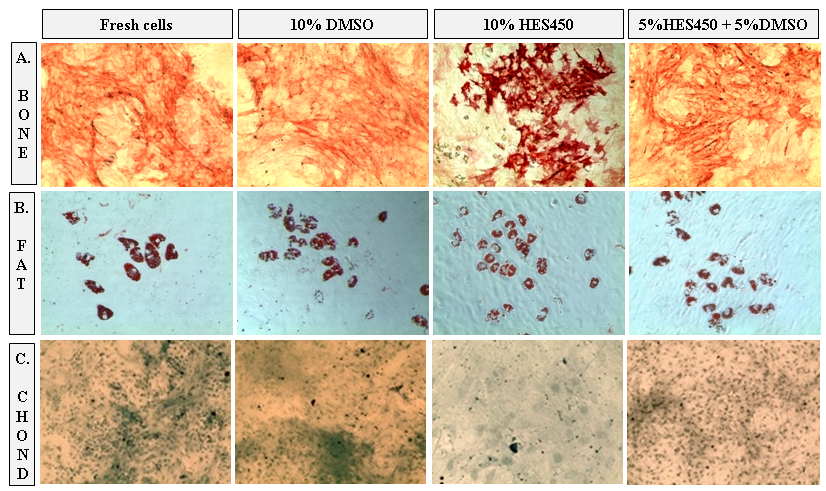

Supplement: Additional file 3 — Figure S1. Differentiation capacity of rat MSCs after cryopreservation. Qualitative ALP-staining of MSCs after 14 days osteogenic differentiation. Magnification 20X (A). Oil red O staining of MSCs after 14 days in adipogenic differentiation medium. Magnification 40X (B). Chondrogenic staining of differentiated MSCs cells after 14 days. Magnification 20X (C). [file 1472-6750-12-49-S3.tiff]

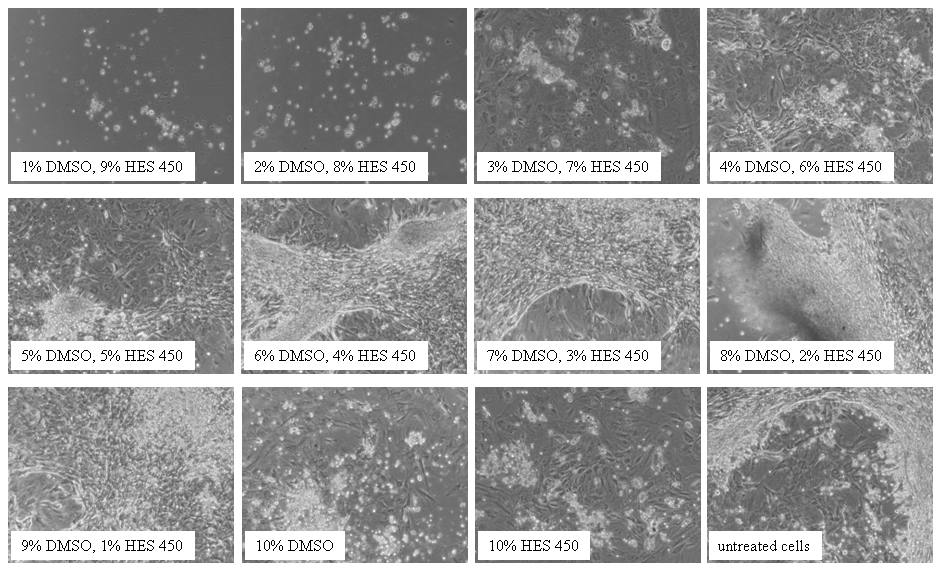

Supplement: Additional file 4 — Figure S3. Morphology of cryopreserved rat MSC during osteogenesis. Cellular morphology of cryopreserved rat MSC after 14 days in osteogenic differentiation medium. [file 1472-6750-12-49-S4.tiff]
